# Functional MYB transcription factor gene *HtMYB2* is associated with anthocyanin biosynthesis in *Helianthus tuberosus* L

**DOI:** 10.1186/s12870-020-02463-8

**Published:** 2020-06-01

**Authors:** Jieming Gao, Xuemei Sun, Yuan Zong, Shipeng Yang, Lihui Wang, Baolong Liu

**Affiliations:** 1 Academy of Agriculture and Forestry Sciences, Qinghai University, Xining, 810016 China; 2 Qinghai Province Key Laboratory of Vegetable Genetics and Physiology, Xining, 810016 China; 3 Key Laboratory of Adaptation and Evolution of Plateau Biota, Northwest Institute of Plateau Biology, Chinese Academy of Sciences, Xining, 810001 China

**Keywords:** *Helianthus tuberosus* L., Tuber epidermis color, Anthocyanin, MYB

## Abstract

**Background:**

Tuber color is an important trait for *Helianthus tuberosus* L. (Jerusalem artichoke). Usually, purple tubers with high anthocyanin content are more nutritious than white tuber. But, the molecular mechanism underlying it is unknown.

**Results:**

In the current study, high-throughput RNA-sequencing was used to compare the transcriptomes between plants with tubers with red or white epidermis. Compared with the white-skinned tubers of cultivar QY3, anthocyanin biosynthesis structural genes had greater expression in the red-skinned tubers of cultivar QY1, indicating that the anthocyanin biosynthesis pathway was activated in ‘QY1’; quantitative PCR confirmed this difference in expression. *HtMYB2* (Unigene44371_All) was the only MYB transcription factor, homologous to the MYB transcription factor regulating anthocyanin biosynthesis, expressed in the red tuber epidermis of ‘QY1’. The anthocyanin concentration in the root, stem, leaf, flower, and tuber epidermis of ‘QY1’ was higher than in ‘QY3’, especially tuber epidermis. Correspondingly, *HtMYB2* had greater expression in these tissues of ‘QY1’ than in ‘QY3’. The expression of *HtMYB2* was associated with anthocyanin accumulation in the different tissues. Overexpression of *HtMYB2* activated the anthocyanin biosynthesis pathway, accumulating the pigment in leaves of transgenic tobacco, supporting the model that *HtMYB2* regulated anthocyanin biosynthesis. Further experiments found that *HtMYB2* had the same coding sequence and genomic sequence in ‘QY1’ and ‘QY3’, but that there were several single nucleotide polymorphisms and one insertion–deletion (indel) mutation of 21 nucleotides in the promoter region between the two alleles. The deletion of three nucleotides “AAA” made the promoter of ‘QY1’ predicted to contain one more possible promoter region. A specific primer, based on the indel, could differentiate between cultivars with red or white tuber epidermis. The genetic variation in *HtMYB2* was associated with the tuber skin color in a natural population.

**Conclusions:**

RNA-seq can successfully isolate the candidate gene (*HTMYB2*) controlling anthocyanin biosynthesis in purple epidermis of Jerusalem artichoke tuber. *HTMYB2* can regulate anthocyanin biosynthesis in plants and is closely related to the formation of purple phenotype in tubers. This study should be useful in understanding the genetic mechanism underlying different tuber skin colors and in breeding new *H. tuberosus* cultivars with different tuber skin colors.

## Background

*Helianthus tuberosus* L., Jerusalem artichoke or topinambour, belongs to the Asteraceae family and is native to North America [1]. The tubers of *H. tuberosus* are rich in fructans, making them a good source of inulin [2], bioethanol [3], and animal feed [4]. Usually, the tuber skin color of *H. tuberosus* is white, although some cultivars produce tubers with pink, purple or red epidermis. Tuber color is an important parameter by which to differentiate between cultivars of *H. tuberosus*, the color difference being due mainly to qualitative and quantitative differences in anthocyanins [5, 6].

Anthocyanins are the major class of water-soluble pigments found in plants, and belong to the flavonoid polyphenols [7]. The metabolic pathway of anthocyanin is relatively well documented in model plants [8, 9]. The structural genes for anthocyanin biosynthesis include *chalcone synthase* (*CHS*), *chalcone isomerase* (*CHI*), *flavonoid-3-hydroxylase* (*F3H*), *flavonoid-3′-hydroxylase* (*F3’H*), *flavonoid-3′,5′-hydroxylase* (*F3’5’H*), and *dihydroflavonol 4-reductase* (*DFR*), *anthocyanidin synthase* (*ANS*) [10].

Generally, expression of the structural genes of anthocyanin biosynthesis is regulated by transcription factors, namely WD40, bHLH and R2R3-MYB proteins. The transcription factors regulate the expression of structural genes by forming trimer complexes and binding with the promoters of the structural genes [11]. Allelic variation in the transcription factor genes has been associated with phenotypic variation related to anthocyanin biosynthesis. The transcription factor encoded by the *R3MYB* gene of dahlia, another member of the Asteraceae, has a domain typical of an MYB gene, which is expressed in colorful dahlia cultivars and can activate the anthocyanin synthesis pathway [12]. The *CtMYB13* transcription factor from safflower (an Asteraceae member) is an important transcription factor regulating the structural genes of the safflower flavonoid biosynthesis pathway [13]. The genetic mechanism of anthocyanin pigment formation has been studied thoroughly in a number of plants, but little is known of the mechanisms involved in *H. tuberosus.*

High-throughput sequencing (RNA-Seq) technology has become a low-cost and highly efficient tool, which can be used to quickly obtain transcripts of various plant types [14, 15]. Due to the large amount of information available on the anthocyanin biosynthesis pathway in plants, the genes related to anthocyanin biosynthesis can be quickly identified though transcriptome analysis in plants, even without the availability of the corresponding genome sequence. Through transcriptome sequencing, the gene encoding the MYB transcription factor *LrAN2* was isolated from *Lycium barbarum*, and those encoding bHLH transcription factors *TaMYC1* and *ThMYC4E* were isolated from wheat without genome sequence information, and further experiments confirmed that they were the key genes responsible for black fruit, purple grain and blue grain traits in the corresponding species, respectively [16–18].

For *H. tuberosus*, there have been no reports on the identification of the key genes responsible for traits associated with anthocyanin biosynthesis, and only a few of the genes related to anthocyanin biosynthesis have been isolated based on homolog cloning. In the current study, RNA-Seq was employed to compare the transcript differences between cultivars with white or red tuber epidermis, and the candidate key genes were isolated to perform function verification, and to understand the relationship between allelic and phenotypic variation.

## Results

### Transcriptome analyses of two *H. tuberosus* cultivars

**Fig. 1:**
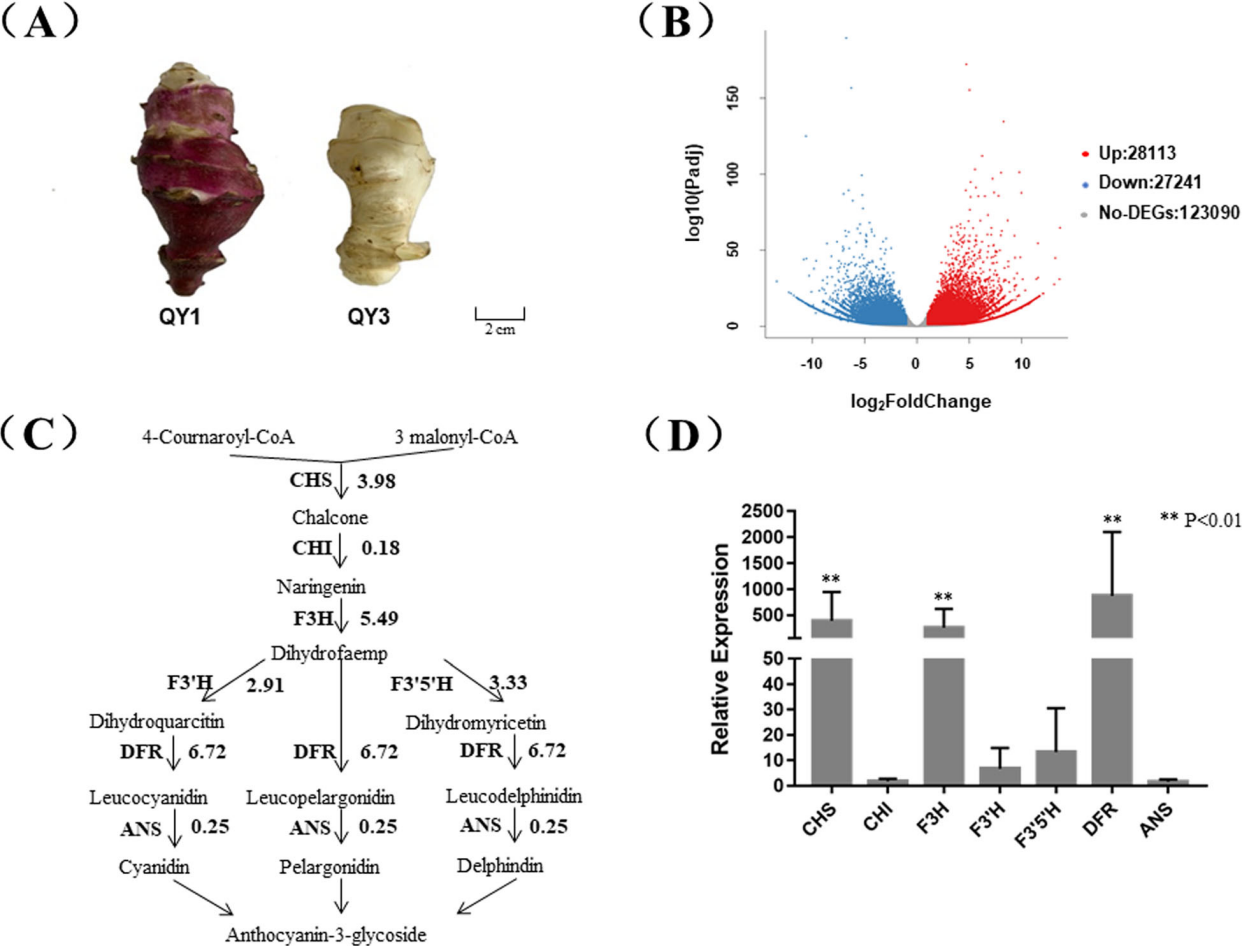
The transcript comparison in *H. tuberosus* QY1 and QY3, with purple and white tuber epidermis. **a** The phenotype of tubers of QY1 and QY3. ‘QY1’ and ‘QY3’ are *H. tuberosus* cultivars bred by Qinghai Academy of Agricultural and Forestry Sciences (Xining 810,000, China). The tuber epidermis of ‘QY1’ is red, whereas that of ‘QY3’ is white. **b** The volcano distribution map of differentially expressed genes in the tuber epidermis of QY1 and QY3 on RNA-seq experiments. The genes were classified into three classes. Red genes are up-regulated if gene expression in the tuber epidermis of QY1 was larger than QY3. Blue genes are down-regulated that gene expression of QY3 was larger than QY1. Gray genes are not differentially expressed. The X-axis represents Log2(Fold change). The Y-axis represents the value of -log10 (Padj). **c** The expression differences of structural genes in the anthocyanin biosynthesis pathway based on RNA-seq experiment. Arrow showed the metabolic stream, abbreviation left or upward arrows represent the genes catalyzing the progress, the number represent the average log2foldchange of the transcript level in the tuber epidermis of QY1 against QY3. **d** Relative transcript level of the structural genes of anthocyanin biosynthesis in the tuber epidermis of QY1 compared with QY3 based on qPCR. The Unigene33222_All, CL2784.Contig1_All, CL9203.Contig4_All, CL13771.Contig2_All, CL13771.Contig1_All, CL9517.Contig40_All, CL13383.Contig1_All were selected to design primer for qPCR experiment of *CHS*, *CHI*, *F3H*, *F3’H*, *F3’5’H*, *DFR* and *ANS*

Based on the Hiseq 2000 platform, RNAs from the tuber epidermis of QY1 and QY3 were sequenced (Fig. 1a). A total of 50 Gb clean data was obtained from three samples from each of the two cultivars after filtering (Table S1). Using Trinity software, 197,769 unigenes were assembled. A total of 55,354 unigenes were differentially expressed, of which 28,113 unigenes were up-regulated, and 27,241 unigenes were down-regulated (Fig. 1b). The unigenes identified as being homologous to the genes involved in anthocyanin synthesis were selected, and their FPKM values for each cultivar were aggregated. None of the anthocyanin biosynthesis structural genes had lower expression levels in ‘QY1’ than in ‘QY3’ (Fig. 1c), and the transcript levels of the key structural genes *CHS*, *CHI*, *F3H*, *F3’H*, *F3’5’H*, *DFR* and *ANS* were higher in ‘QY1’ than in ‘QY3’. The fold up-regulation of structural genes in ‘QY1’, relative to ‘QY3’, reached 3.98, 0.18, 5.49, 2.91, 3.33, 6.71 and 0.25, respectively (Table S2). Unigene33222_All, CL2784.Contig1_All, CL9203.Contig4_All, CL13771.Contig2_All, CL13771.Contig1_All, CL9517.Contig40_All, CL13383.Contig1_All were selected to design primer for qPCR experiment of *CHS*, *CHI*, *F3H*, *F3’H*, *F3’5’H*, *DFR* and *ANS*. The qPCR results also confirmed these findings, though the numerical values differed somewhat with respect to some genes (Fig. 1d). Therefore, the activation of the anthocyanin biosynthesis structural genes appeared to be the cause of the red tuber trait in ‘QY1’ but not ‘QY3’. As with the up-regulation of expression of the structural genes in ‘QY1’, the genes encoding transcription factors MYB and bHLH exhibited greater expression levels in ‘QY1’ than in ‘QY3’ (Table S2). Considering that the structural genes were regulated by the transcription factors, and that the MYB transcription factor could induce expression of the bHLH transcription factor [19]. *HtMYB2* (Unigene44371_All) should be the key gene responsible for the red tuber skin color trait in *H. tuberosus*.

### Molecular characteristics of HtMYB2

**Fig. 2:**
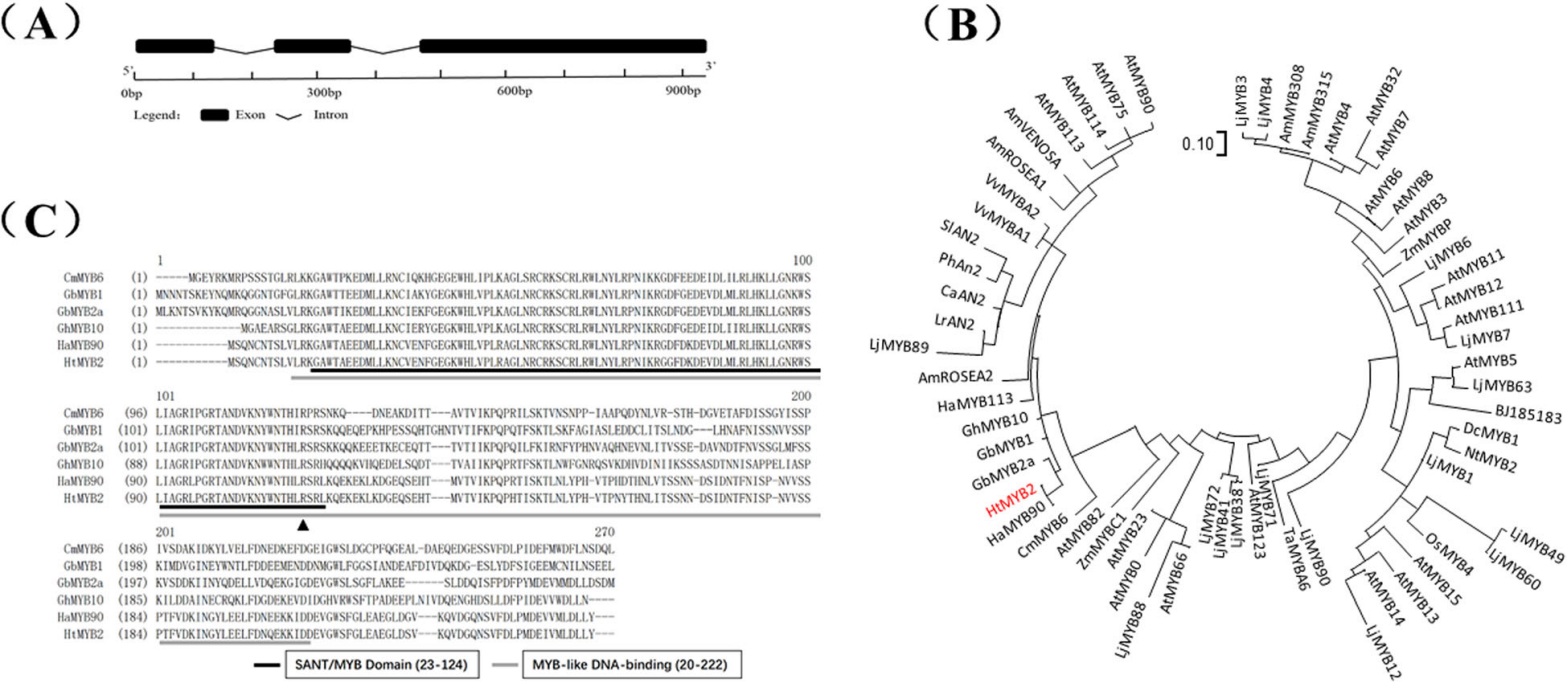
The molecular characteristics of *HtMYB2*. **a** The gene structure of *HtMYB2*. The black squares represent exons, and the black lines represent introns. **b** The phylogenetic tree of MYB proteins regulating anthocyanin biosynthesis. GbMYB2a:BAP47698.1; GbMYB1:BAJ17661.1; GhMYB10:AAK19615.1; HaMYB113:XP_022033329.1; AmROSEA2:ABB83827.1; LjMYB89:AFK35838.1; LrAN2:QCS14086.1; CaAN2:NP_001311547.1; PhAN2:AB982128.1; SlAN2:FJ705319.1; VvMYBA1:ABD72954.1; VvMYBA2:BAD18978.1; AmROSEA1:ABB83826.1; AmVENOSA:ABB83828.1; AtMYB113:NM_105308.2; AtMYB114:NM_001334235.1; AtMYB75: ABB03879.1; AtMYB90:NP_176813.1; LjMYB3:ALU11262.1; LjMYB4:ALU11258.1; AmMYB308:ABI26190.1; AmMYB315:AAV70655.1; AtMYB4:NP_195574.1; AtMYB32:NP_195225.1; AtMYB7:OAP08362.1; AtMYB6:NP_192684.1; AtMYB8:BAE99960.1; AtMYB3:NP_564176.2; ZmMYBP:AAL90641.1; LjMYB6:E5L8F7.1; AtMYB11:NP_191820.1; AtMYB12:ABB03913.1; AtMYB111:EFH41988.1; LjMYB7:AKV17427.1; AtMYB5:NP_187963.1; LjMYB63:ALU11252.1; BJ185183:XP_024388078.1; DcMYB1:XP_017224603.1; NtMYB2:XP_009629052.1; LjMYB1:AFK36130.1; LjMYB49:ALU11251.1; LjMYB60:XP_019055104.1; OsMYB4:XP_015633465.1; AtMYB15:NP_188966.1; AtMYB13:NP_172108.1; AtMYB14:XP_002881162.1; LjMYB12:3760052; LjMYB90:ALU11257.1; TaMYBA6:BAH36890.1; LjMYB71:ALU11256.1; AtMYB123:ABK28720.1; LjMYB38:BAG12893.1; LjMYB41:BAG12894.2; LjMYB72:BAG12893.1; AtMYB66:NP_196979.1; LjMYB88:ALU11254.1; AtMYB0:AAL01241.1;AtMYB23:CDY44571.1; ZmMYC1:1613412E; AtMYB82:AAF14064.1; CmMYB6:AKP06190.1; HaMYB90:XP_022033410.1; **c** The amino acid alignment of HtMYB2 and the closest anthocyanin-related MYB transcription factors. CmMYB6: AKP06190.1; GbMYB1: BAJ17661.1; GbMYB2a: BAP47698.1; GhMYB10: AAK19615.1; HaMYB90: XP_022033410.1. The triangle repesent the site of the different amino acid of HtMYB2 from QY1 and QY3. The amino acid “R” in QY1 was “K” in QY3

Based on transcriptome information, the genomic and coding sequences (CDSs) of *HtMYB2* were isolated from ‘QY1’ and ‘QY3’. The genomic sequence of *HtMYB2* from ‘QY1’ and ‘QY3’ contained 1066 bp and 1068 bp, respectively, while the length of the coding sequences were same. *HtMYB2* contained three introns and two exons (Fig. 2a). Although two nucleotide differences existed in the third exon of the CDSs of ‘QY1’ and ‘QY3’, only one amino acid difference was found in the translated sequence (Fig. 2c). The phylogenetic tree of the MYB transcription factors showed that HtMYB2 was similar to the MYB transcription factors controlling the traits associated with anthocyanin biosynthesis in same species, including members of the *Asteraceae*, the *Solanaceae*, and the *Brassicaceae* (Fig. 2b). Compared with the most similar MYB transcription factors CmMYB6 (from *Chrysanthemum morifolium*, Asteraceae), GbMYB1, GbMYB2a (from *Gynura bicolor*, Asteraceae), GhMYB10 (from *Gossypium hirsutum*, Malvaceae), and HaMYB90 (from *Helianthus annuus*, Asteraceae), HtMYB2 contained the intact MYB-like binding domain (Fig. 2c), which is important to carry out the function of the MYB transcription factor in regulating anthocyanin biosynthesis. This implied that *HtMYB2* should have the function for regulating anthocyanin biosynthesis.

### Overexpression of HtMYB2 induces anthocyanin biosynthesis in tobacco

**Fig. 3:**
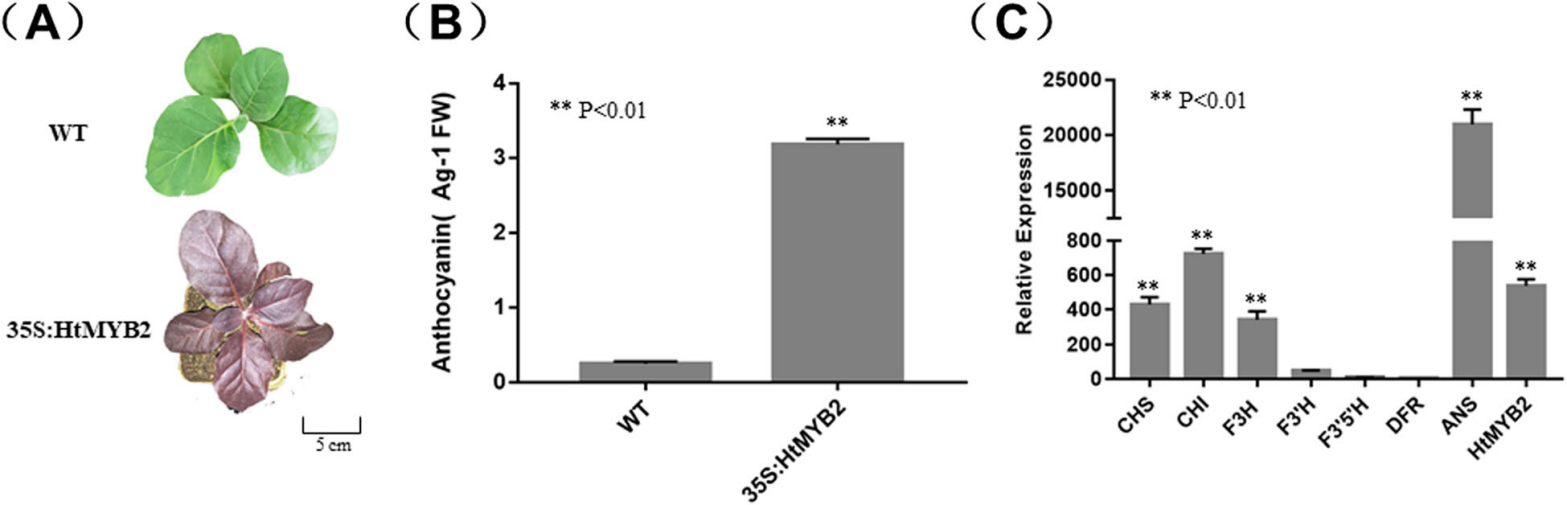
The relative anthocyanin contents and transcript level of anthocyanin biosynthesis pathway in the leaves of the HtMYB2-overpression transgenic tobacco and wild type (WT) 3 weeks after germinating. (A) The relative anthocyanin content of transgenic lines and WT. (B) Relative transcript level of *HtMYB2* and the structural genes relative to anthocyanin biosynthesis in leaves of transgenic tobacco and WT.

The pJAM1502:HtMYB2 plasmid was transferred into *Agrobacterium tumefaciens* strain LBA4404 by the freeze-thaw method. The *Agrobacterium*-mediated leaf disk transformation method was performed to obtain transgenic tobacco. For further experiments, the T3 family lines carrying objective gene without the separation were used. The positive transgenic lines exhibited deep purple leaves (Fig. 3a), and the relative anthocyanin concentration of the transgenic lines was much higher than that of the wild type (Fig. 3b). The qPCR experiment showed that the expression levels of the anthocyanin synthesis-related structural genes and of *HtMYB2* were up-regulated in the transgenic lines (Fig. 3c). These results showed that *HtMYB2* can activate anthocyanin biosynthesis by acting as a MYB transcription factor in tobacco.

### The relation between the transcript abundance of *HtMYB2* and anthocyanin concentration in different tissues

**Fig. 4:**
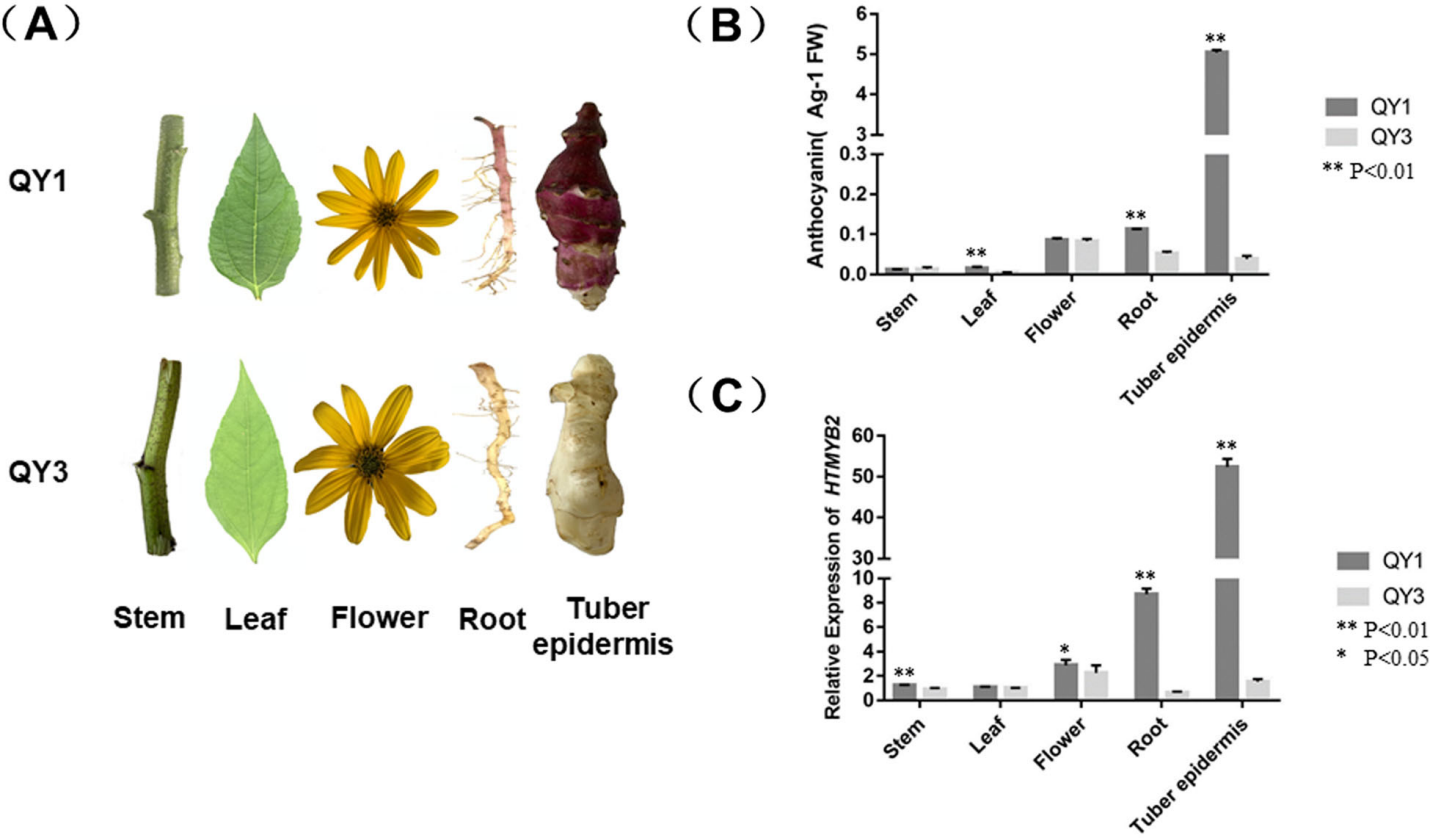
The anthocyanin content and the transcript level of *HtMYB2* in different tissues of QY1 and QY3. **a** The phenotype of stem, leaf, flower, root and tuber of QY1 and QY3. **b** The anthocyanin content in different tissues of QY1 and QY3. **c** The relative transcript level of *HtMYB2* and the structural genes relative to anthocyanin biosynthesis in different tissues

Visually, the root and tuber epidermis of ‘QY1’ were significantly redder than those layers of ‘QY3’, whereas there was little phenotypic difference among stem, leaf and flower from the two cultivars (Fig. 4a). Correspondingly, the anthocyanin concentration of tuber peel and root of ‘QY1’ was significantly higher than that of ‘QY3’, while there was no significant difference in anthocyanin concentration of stem, leaf or flower between the two cultivars (Fig. 4b). The expression of *HtMYB2* was consistent with the anthocyanin concentrations. The tissue with highest *HtMYB2* expression was the tuber epidermis of ‘QY1’, followed by the root of ‘QY1’ (Fig. 4b), whereas the other tissues of ‘QY1’ and all the tissues of ‘QY3’ showed little expression of *HtMYB2*. Each treatment was replicated three times.

### Allelic variation of HtMYB2 in natural populations of *Helianthus tuberosus* L

*HtMYB2* exhibited clear differences in expression level in the tuber epidermis between ‘QY1’ and ‘QY3’. The promoter was isolated from *HtMYB2* from each cultivar, using TAIL-PCR, in an attempt to explain the difference in expression of *HtMYB2* between the two cultivars. The promoter from ‘QY1’ had three possible promoter regions, based on the promoter prediction software BDPG, while ‘QY3’ contained only two (Table S3). The deletion of three nucleotides “AAA” in ‘QY1’ caused the difference in the promoters of the two cultivars.

**Fig. 5:**
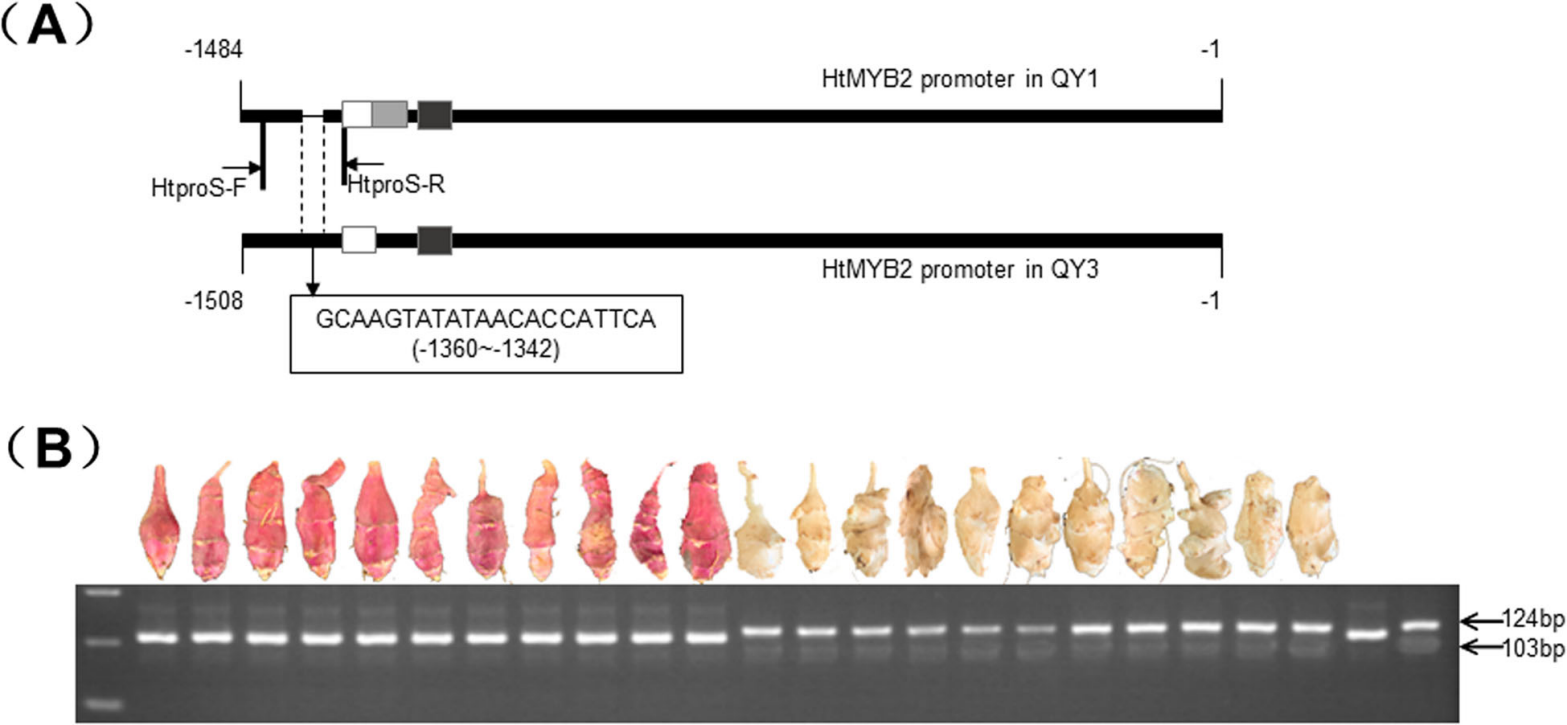
The allelic variation of *HtMYB2* in natural population. **a** The difference in the promoter region of *HtMYB2*. The sequence in the box indicates that 21 bp was inserted into the promoter of QY3. White, gray and black boxes were the three active regions of promoters predicted by BDPG software (Table S3). The gray box only existed in the promoter of QY1. Arrow shows the site of specific primer HtproS. **b** The electrophoresis of PCR production of partial materials with the diagnostic primers HtproS. The target strips of purple varieties were 103 bp, and the white varieties were 124 bp

Compared with the promoter of QY3, 21 bp were deleted in the region − 1360 to − 1342 of the promoter of QY1 (Fig. 5a). Based on the indel difference between the two promoters, the diagnostic primer HtproS was designed to differentiate the *HtMYB2* from ‘QY1’ and ‘QY3’. The length of the amplification fragment from ‘QY1’ was 103 bp, whereas that of the ‘QY3’ amplification fragment was 124 bp (Fig. 5a). This primer pair can effectively distinguish *HtMYB2*-QY1 from *HtMYB2*-QY3 (Fig. S1). In 180 selected individual plants, 90 individuals with red-skinned tubers carried the genotype *HtMY23*-QY1, while 90 individuals with white-skinned tubers carried the genotype *HtMYB2*-QY3 (Fig. 5b) (Table S4). The results showed that allelic variation in *HtMYB2* was consistent with tuber skin color in *H. tuberosus****.***

## Discussion

In this study, we isolated a MYB transcription factor, *HtMYB2*, from *H. tuberosus* and explored its function in relation to anthocyanin biosynthesis and the red tuber skin color trait.

### HtMYB2 is a functional MYB transcription factor gene regul*ating anthocyanin biosynthesis*

*HtMYB2* has the character of a functional MYB transcription factor. It has two introns and three exons. The protein encoded by HtMYB2 contained an intact MYB-like DNA-binding domain and a SANT domain, which played an important role in the regulation of anthocyanin biosynthesis. In the phylogenetic tree, HtMYB2 was closest to the MYB transcription factors GbMYB2 and CmMYB6 [20]. GbMYB2 encodes a R2R3 MYB transcription factor and regulates anthocyanin biosynthesis in leaves of *G. bicolor,* another member of the Asteraceae [21]. CmMYB6 from *C. morifoium*, also a member of the Asteraceae, could induce an approximately 34-fold increase in transcription of *CmDFR*, with the help of *MrbHLH* [22]. Most importantly, the overexpression of exogenous *HtMYB2* in tobacco activated the expression of the endogenous structural genes related to anthocyanin biosynthesis, and increased the anthocyanin concentration in the tobacco leaves. The structural genes of anthocyanin biosynthesis which was mainly up-regulated were different between in QY1 and in transgenic tobacco. It should be due to the genetic variation in the promoter of the structural genes in different species. All of these results implied that *HtMYB2* was a functional MYB transcription factor regulating anthocyanin biosynthesis.

### The HtMYB2 function was associated with t*he tuber epidermis color trait*

In transcriptome analysis, expression of the structural genes of anthocyanin biosynthesis was activated in the tuber epidermis of ‘QY1’, a finding which was also confirmed by qPCR. As is known, expression of the anthocyanin structural genes is regulated by MYB and bHLH transcription factors, with the MYB transcription factors inducing the expression of the bHLH transcription factor. In fact, *HtMYB2* was the only MYB transcription factor, regulating anthocyanin biosynthesis, expressed at a high level in the *H. tuberosus* ‘QY1’ tuber epidermis, where anthocyanins accumulated, indicating that *HtMYB2* was involved in anthocyanin biosynthesis in the tuber epidermis of ‘QY1’. Moreover, the transcript abundance of *HtMYB2* was consistent with the anthocyanin concentrations in different tissues. Anthocyanins were detected in only the root and tuber organs of ‘QY1’, which also contained higher transcript abundance of *HtMYB2* than the other organs. In the promoter of the two alleles, although HtMYB2promoter-QY3 inserts 21 bp sequences. However, in the prediction results of promoter functional area, HtMYB2promoter-QY1 has more than one functional area with a score of 0.83 from – 1300 bp to – 1250 bp, which is likely that these differences lead to *HtMYB2* failure to activate the anthocyanin biosynthesis pathway in white varieties. Two alleles, *HtMYB2*-QY1 and *HtMYB2*-QY3, were present in the *H. tuberosus* cultivars ‘QY1’ and ‘QY3’, respectively. The allelic variation was associated with the tuber epidermis color in natural populations of *H. tuberosus* segregating for tuber skin color trait; *HtMYB2*-QY1 was linked to the red tuber epidermis trait, whereas *HtMYB2*-QY3 was associated with the white tuber epidermis trait. All in all, *HtMYB2* appears to be the key gene responsible for the red tuber epidermis trait in *H. tuberosus*.

## Conclusion

In the present study, *HtMYB2* was isolated from *H. tuberosus* by RNA-seq. It had the same intron and exon number and the same functional domain as other MYB transcription factors which had been shown to regulate anthocyanin biosynthesis in other plants. *HtMYB2* was close to such functional MYB transcription factors in a phylogenetic tree. Overexpression of *HtMYB2* induced anthocyanin biosynthesis in tobacco. Though *HtMYB2* had similar coding sequences in cultivar QY1 with red-skinned tubers and cultivar QY3 with white-skinned tubers, the transcript abundance of *HtMYB2* was significantly higher in the tuber epidermis of ‘QY1’ than in ‘QY3’. *HtMYB2* transcripts were detected in only the root and tuber epidermis of ‘QY1’. Promoter differences were associated with differences in transcript abundance in *HtMYB2* between ‘QY1’ and ‘QY3’. Allelic variation in the *HtMYB2* gene was closely associated with tuber color in a natural population. All results implied that *HtMYB2* is a functional MYB transcription factor, regulating anthocyanin biosynthesis in *H. tuberosus*, and playing an important role in determining the red tuber epidermis trait, which should be useful information for breeding new cultivars of *H. tuberosus* with different tuber colors.

## Methods

### Plant materials

‘QY1’ and ‘QY3’ are *H. tuberosus* cultivars bred by Qinghai Academy of Agricultural and Forestry Sciences (Xining 810,000, China). The tuber epidermis of ‘QY1’ is red, whereas that of ‘QY3’ is white (Fig. 1a). All materials were planted and stored in the Institute of Horticulture, Qinghai Academy of Agricultural and Forestry Sciences (E101°45′08.15″, N36°43′32.06″). The library label of these samples were recorded in Table S4. The *Nicotiana tabacum* cultivar Samsun was chosen as a transformation plant. *Nicotiana tabacum* (Samsun) was given by Professor Cathie Martin from John Innes Centre, and stored now in Northwest Plateau Institute of Biology, Chinese Academy of Sciences. No permission was required in collecting the plants. In this study, Yuan Zong was responsible for the planting and identification of these samples.

### Transcriptome analysis

Tuber epidermis samples of ‘QY1’ and ‘QY3’ were collected in triplicate and used as the source material from which the transcriptomes were generated. Each of the three transcriptomes was generated from a different sample of ‘QY1’ and ‘QY3’. The cDNA libraries of tuber epidermis were created according to the descrition of instrument sample requirements for mRNA-Seq sample preparation (Illumina Inc., San Diego, CA, USA). The cDNA library products were sequenced by Illumina paired-end sequencing technology with read lengths of 150 bp, and they were sequenced on the Illumina HiSeq 2000 platform by Novogene with three repeats. Before assembly, original reads were filtered to obtain high-quality clean reads. Sequences with ambiguous bases (denoted with > 5% ‘N’ in the sequence trace), low-quality reads (the rate of reads with a quality value ≤10 was more than 20%) and reads with adapters should be removed. After puritfying all reads, Trinity was used to assembly the high-quality reads, with default parameters to construct unique consensus sequences [23]. The expression levels of every unigene was calculated based on the FPKM (fragments per kilobase of transcript per million mapped reads) values. Difference in Unigenes between purple and white sample transcripts were identified by the Chi-square test, using IDEG6 software [24]. The False Discovery Rate (FDR) method was introduced to determine the threshold *p*-value at FDR ≤ 0.001, with the absolute value of |log2Ratio| ≥ 1 being used as the threshold to determine the significance of the differential expression of unigenes [25]. All Unigenes related to anthocyanin biosynthesis in the Kyoto Encyclopedia of Genes(KEGG) and Genomes(GO) pathways were collected and aligned to the unigenes of the transcriptome, using BlastX with e-value <1e-5 [26]. In order to comparing the relative expression levels of unigenes, the FPKM values of unigenes aligned to genes of the anthocyanin biosynthesis pathway were accumulated together.

### DNA and cDNA preparation

Genomic DNA of Jerusalem artichoke was extracted from 1 g fresh weight tuber [27]. Total RNA was extracted from root, stem, leaf, flower and tuber epidermis of different Jerusalem artichoke organs, using the Trizol method [28]. The synthesis of the first strand of cDNA was carried out according to the manufacturer’s instructions of the First Strand Synthesis Kit of Fastking gDNA Dispelling RT SuperMix (TIANGEN, Beijing, China). The DNA and synthesized cDNA were stored at − 20 °C prior to subsequent gene cloning and qPCR analysis.

### PCR and qPCR analysis

The primers were designed by PRIMER 6.0 (Palo Alto, CA, USA) and synthesized by BGI Biological Technology Co., Ltd. (BGI Company, Beijing, China). The 50 μl reaction volume included 25 μl 2× Unique HiQTM PCR Buffer, 0.5 μl Pfu DNA Polymerase (Thermo Fisher Science, Beijing, China), 0.5 μl 20 pmol primers each, and 0.5 μl cDNAs, and were made up to volume with dd H_2_O. The PCR procedure was: 98 °C for 2 min, 98 °C for 10 s, 53 °C for 30 s, 72 °C for 2 min, for 30 cycles, followed by 72 °C for 10 min, and then storage at 4 °C. The PCR products were detected by 1% agarose gel electrophoresis and photographed by a gel imaging analyzer (Tanon, Shanghai, China). All primers used in this research are listed in Table S5.

In order to analyze the transcription level of genes related to anthocyanin synthesis, real-time fluorescence quantitative PCR (qPCR) was performed on an Applied Biosystems QuantStudio® 3 Real-Time PCR System (Thermo Fisher Company, Beijing, China). The fusion curve was analyzed to confirm the specificity of the amplification. The reaction mixture (20 μL): 2× SYBR Green 10 μL, ddH_2_O 7.8 μ L, primers 0.6 μl each, and cDNA template 1 μL (about 100 ng/μL). The PCR thermal cycle consisted of four steps as follows: pre denaturation at 95 °C for 15 min, denaturation at 95 °C for 10 s, annealing at 60 °C for 20 s, and extension at 72 °C for 30 s, with 40 cycles in total. Fluorescence signals were collected at the 60 °C annealing stage to obtain circulating *C*_T_ values for different genes. The data were analyzed using the 2 ^- ΔΔ^C_T_ method [29].

### Bioinformatics analysis

The online software of ExPASY translate (https://web.expasy.org/translate/) was used to predict the protein. BlastP (https://blast.ncbi.nlm.nih.gov/blast.cgi) in NCBI was used to predict the conserved protein regions. The neighbor-joining method was used to construct phylogenetic trees with default parameters based on the software MEGA6 (http://www.megasoftware.net/mega6/faq.html) [30]. BDPG (http://www.fruitfly.org/seq_tools/promoter.html) was used to predict the functional domain in promoter.

### Overexpression of HtMYB2 in tobacco

The overexpression vector for tobacco transformation was based on the pJAM1502 binary vector, which contains a double CaMV35S promoter [31]. The pJAM1502: HtMYB2 construct was achieved using the Gateway cloning Kit (Invitrogen, Carlsbad, CA, USA). Binary vectors were electroporated into *Agrobacterium tumefaciens* strain GV3101. Tobacco (*Nicotiana tabacum*) transformation was carried out using a leaf disc transformation method [32]. Transgenic shoots were grown on selective medium containing 3% (w/v) sucrose, 0.7% (w/v) MS (Murashige and Skoog), 0.7% (w/v) agar, 1.0 mg/mL 6-benzylaminopurine(6-BA), 1.0 mg/mL 1-naphthaleneacetic acid(NAA), 300 mg/L Hygromycin and 150 mg/L kanamycin. These transgenic shoots were transferred to the greenhouse under long-day light conditions (16 h light/8 h dark) after 1 month. Significant differences were determined using analysis of variance (ANOVA) and Tukey’s honestly significant difference (HSD) test, where *P* < 0.05 was considered to be significant. All data were analyzed using SPSS software (IBM, USA).

### Anthocyanin measurement

Anthocyanins were extracted by the method for “total monomeric anthocyanin pigment content of fruit juice, beverages, natural colors, and wines” (AOAC Official Method 2005.02). The absorbances (A) at 530 nm and 657 nm (expressed as ΔA g^− 1^ fresh weight was measured using a spectrophotometer (Beijing General Analysis Company, Beijing, China). The relative content of anthocyanin in the extract was calculated as [ΔA = A_530_ – (0.25 × A_657_)], and the effects of chlorophyll and its degradation products on the absorbance results were corrected [33, 34].

### Genotyping of a natural population of *Helianthus tuberosus*

The promoter sequences of *HtMYB2* were isolated from ‘QY1’ and ‘QY3’, based on thermal asymmetric interlaced (TAIL)-PCR [35]. According to the nucleotide sequence differences between the promoters of *HtMYB2* of ‘QY1’ and ‘QY3’, a polymorphic PCR marker HtproS was designed to distinguish between ‘QY1’ and ‘QY3’ (Table S5). The allelic variation in *HtMYB2* was identified in the natural population of *H. tuberosus*. One hundred eighy Jerusalem artichoke materials from different regions, and DNA extraction for backup (Table S4).

## Supplementary information

**Additional file 1: FigureS1.** Development of the diagnostic primer HTproS for amplifying the two different alleles of HTMYB2 (QY1 and QY3). The fragments yielded by the marker were either 124 bp or 103 bp, which were indicative of promoter in QY1 and QY3, respectively.

**Additional file 2: Table S1.** The statistic of sequencing, filtering and assembling in transcriptome analysis.

**Additional file 3: Table S2.** The expression level of structural and regulation genes in the anthocyanin biosynthesis pathway.

**Additional file 4: Table S3.** The promoter prediction of HtMYB2.

**Additional file 5: Table S4.** The origin of the *Helianthus tuberosus* and their MYB genotype examined using HtproS marker.

**Additional file 6: Table S5.** Oligo nucleotide primers used in this work.

## Data Availability

*HtMYB2* has been uploaded to NCBI, Genebank: MN887536 (Released on May 11, 2020). The transcriptomic data has been successfully uploaded to NCBI (http://www.ncbi.nlm.nih.gov/bioproject/605502), Submission ID: SUB6924386; BioProject ID: PRJNA605502. All data generated or analyzed during this study are included within the article and its additional files.

## Abbreviations

CHS: Chalcone synthase
CHI: Chalcone isomerase
F3H: Flavonoid-3-hydroxylase
F3’H: Flavonoid − 3′- hydroxylase
F3’5’H: Flavonoid-3′,5′-hydroxylase
DFR: Dihydroflavonol 4-reductase
ANS: Anthocyanidin synthase
CDSs: Coding sequences
WT: Wild type
FDR: False discovery rate
bHLH: Basic Helix-Loop-Helix
qPCR: Quantitative real-time polymerase chain reaction.

## References

[1] Chen FJ, Long XH, Li EZ (2019). Evaluation of Antifungal Phenolics from *Helianthus tuberosus* L. Leaves against Phytophthora capsici Leonian by Chemometric Analysis. Molecules.

[2] Saengkanuk A, Nuchadomrong S, Jogloy S, Patanothai A, Srijaranai S (2011). A simplified spectrophotometric method for the determination of inulin in Jerusalem artichoke (Helianthus tuberosus L.) tubers. Eur Food Res Technol.

[3] Song Y, Wi SG, Kim HM, Bae HJ (2016). Cellulosic bioethanol production from Jerusalem artichoke (Helianthus tuberosus L.) using hydrogen peroxide-acetic acid (HPAC) pretreatment. Bioresour Technol.

[4] Khuenpet K, Jittanit W, Sirisansaneeyakul S, Srichamnong W (2017). Inulin Powder Production from Jerusalem Artichoke (*Helianthus tuberosus* L.) Tuber Powder and Its Application to Commercial Food Products. J Food Process Pres.

[5] Kim HJ, Kim HM, Lee KG, Shin JS, Ahn HJ, Jeong JC, Kwon OK, Nam JH, Lee KT, Jang DS (2014). P-Coumaroyl Anthocyanins from the tuber epidermis of a colored potato Solanum tuberosum L. cv Jayoung. B Korean Chem Soc.

[6] Hamouz K, Lachman J, Pazderu K, Tomasek J, Hejtmankova K, Pivec V (2011). Differences in anthocyanin content and antioxidant activity of potato tubers with different flesh colour. Plant Soil Environ.

[7] Mottaghipisheh J, Ayanmanesh M, Babadayei-Samani R, Javid A, Sanaeifard M, Vitalini S, Iriti M (2018). Total anthocyanin, flavonoid, polyphenol and tannin contents of seven pomegranate cultivars grown in Iran. Acta Sci Pol Technol Aliment.

[8] Zhou LL, Zeng HN, Shi MZ, Xie DY (2008). Development of tobacco callus cultures over expressing Arabidopsis PAP1/MYB75 transcription factor and characterization of anthocyanin biosynthesis. Planta.

[9] Shan X, Li Y, Yang S, Gao R, Zhou L, Bao T, Han T, Wang S, Gao X, Wang L (2019). A functional homologue of Arabidopsis TTG1 from Freesia interacts with bHLH proteins to regulate anthocyanin and proanthocyanidin biosynthesis in both Freesia hybrida and Arabidopsis thaliana. Plant Physiol Biochem.

[10] Xu ZS, Huang Y, Wang F, Song X, Wang GL, Xiong AS (2014). Transcript profiling of structural genes involved in cyanidin-based anthocyanin biosynthesis between purple and non-purple carrot (*Daucus carota* L.) cultivars reveals distinct patterns. BMC Plant Biol.

[11] Zhao L, Gao L, Wang H, Chen X, Wang Y, Yang H, Wei C, Wan X, Xia T (2013). The R2R3-MYB, bHLH, WD40, and related transcription factors in flavonoid biosynthesis. Funct Integr Genomics.

[12] Ohno S, Hosokawa M, Hoshino A, Kitamura Y, Morita Y, Park KI, Nakashima A, Deguchi A, Tatsuzawa F, Doi M, Iida S, Yazawa S (2011). A bHLH transcription factor, DvIVS, is involved in regulation of anthocyanin synthesis in dahlia (Dahlia variabilis). J Exp Bot.

[13] Pahlavani MH, Mirlohi AF, Saeidi G (2004). Inheritance of flower color and spininess in safflower (Carthamus tinctorius L.). J Hered.

[14] Goyer A, Hamlin L, Crosslin JM, Buchanan A, Chang JH (2015). RNA-Seq analysis of resistant and susceptible potato varieties during the early stages of potato virus Y infection. Bmc Genomics.

[15] Wang Y, Sun MA, White AP (1751). RNA-Seq-based transcript structure analysis with TrBorderExt. Methods Mol Biol.

[16] Zong Y, Zhu X, Liu Z, Xi X, Li G, Cao D, Wei L, Li J, Liu B (2019). Functional MYB transcription factor encoding gene AN2 is associated with anthocyanin biosynthesis in Lycium ruthenicum Murray. BMC Plant Biol.

[17] Zong Y, Xi X, Li S, Chen W, Zhang B, Liu D, Liu B, Wang D, Zhang H (1645). Allelic variation and transcriptional isoforms of wheat TaMYC1 gene regulating anthocyanin synthesis in pericarp. Front Plant Sci.

[18] Li N, Li S, Zhang K, Chen W, Zhang B, Wang D, Liu D, Liu B, Zhang H (2017). ThMYC4E, candidate Blue aleurone 1 gene controlling the associated trait in *Triticum aestivum*. PLoS One.

[19] Zong Y, Li S, Xi X, Cao D, Wang Z, Wang R, Liu B (2019). Comprehensive Influences of Overexpression of a MYB Transcriptor Regulating Anthocyanin Biosynthesis on Transcriptome and Metabolome of Tobacco Leaves. Int J Mol Sci.

[20] Yan H, Mengling L, Silan D (2019). Ectopic Expression of Multiple Chrysanthemum (*Chrysanthemum x morifolium*) R2R3-MYB transcription Factor Genes Regulates Anthocyanin Accumulation in Tobacco. Genes.

[21] Yasuhiro S, Kazuhiro M, Mika K, Koichiro S (2010). Isolation of anthocyanin-related MYB gene, *GbMYB2*, from *Gynura bicolor* leaves. Plant Biotechanology.

[22] Xiang L, Liu X, Li H, Yin X, Grierson D, Li F, Chen K (2019). CmMYB7, an R3 MYB transcription factor, acts as a negative regulator of anthocyanin biosynthesis in chrysanthemum. J Exp Bot.

[23] Haas BJ, Papanicolaou A, Yassour M, Grabherr M, Blood PD, Bowden J, Couger MB, Eccles D, Li B, Lieber M, MacManes MD, Ott M, Orvis J, Pochet N, Strozzi F, Weeks N, Westerman R, William T, Dewey CN, Henschel R, Leduc RD, Friedman N, Regev A (2013). De novo transcript sequence reconstruction from RNA-seq using the trinity platform for reference generation and analysis. Nat Protoc.

[24] Romualdi C, Bortoluzzi S, D'Alessi F, Danieli GA (2003). IDEG6: a web tool for detection of differentially expressed genes in multiple tag sampling experiments. Physiol Genomics.

[25] Zhu D, Hero AO, Qin ZS, Swaroop A (2005). High throughput screening of co-expressed gene pairs with controlled false discovery rate (FDR) and minimum acceptable strength (MAS). J Comput Biol.

[26] Altermann E, Klaenhammer TR (2005). PathwayVoyager: pathway mapping using the Kyoto encyclopedia of genes and genomes (KEGG) database. BMC Genomics.

[27] Cota-Sanchez JH, Remarchuk K, Ubayasena K (2006). Ready-to-use DNA extracted with a CTAB method adapted for herbarium specimens and mucilaginous plant tissue. Plant Mol Biol Rep.

[28] Villa-Rodriguez E, Ibarra-Gamez C, de Los Santos-Villalobos S (2018). Extraction of high-quality RNA from Bacillus subtilis with a lysozyme pre-treatment followed by the Trizol method. J Microbiol Methods.

[29] Nassuth A, Pollari E, Helmeczy K, Stewart S, Kofalvi SA (2000). Improved RNA extraction and one-tube RT-PCR assay for simultaneous detection of control plant RNA plus several viruses in plant extracts. J Virol Methods.

[30] Kumar S, Stecher G, Tamura K (2016). MEGA7: molecular evolutionary genetics analysis version 7.0 for bigger datasets. Mol Biol Evol.

[31] Keadtidumrongkul P, Suttangkakul A, Pinmanee P, Pattana K, Kittiwongwattana C, Apisitwanich S, Vuttipongchaikij S (2017). Growth modulation effects of CBM2a under the control of AtEXP4 and CaMV35S promoters in Arabidopsis thaliana, Nicotiana tabacum and Eucalyptus camaldulensis. Transgenic Res.

[32] Mayo KJ, Gonzales BJ, Mason HS (2006). Genetic transformation of tobacco NT1 cells with agrobacterium tumefaciens. Nat Protoc.

[33] Weiss D, Van Der Luit A, Knegt E, Vermeer E, Mol J, Kooter JM (1995). Identification of endogenous gibberellins in Petunia flowers (induction of anthocyanin biosynthetic gene expression and the antagonistic effect of Abscisic acid). Plant Physiol.

[34] Meng XC, Xing T, Wang XJ (2004). The role of light in the regulation of anthocyanin accumulation in Gerbera hybrida. Plant Growth Regul.

[35] Thanh T, Chi VT, Abdullah MP, Omar H, Napis S (2012). Efficiency of ligation-mediated PCR and TAIL-PCR methods for isolation of RbcS promoter sequences from green microalgae *Ankistrodesmus convolutus*. Mol Biol (Mosk).

