# Supplementary material for: Functional MYB transcription factor gene HtMYB2 is associated with anthocyanin biosynthesis in Helianthus tuberosus L
Source: BMC Plant Biol. 2020 Jun 1;20:247. doi: 10.1186/s12870-020-02463-8 (PMC7268318; doi:10.1186/s12870-020-02463-8)
Supplement: Supplementary file 1 — Additional file 1: FigureS1. Development of the diagnostic primer HTproS for amplifying the two different alleles of HTMYB2 (QY1 and QY3). The fragments yielded by the marker were either 124 bp or 103 bp, which were indicative of promoter in QY1 and QY3, respectively. [file 12870_2020_2463_MOESM1_ESM.docx]

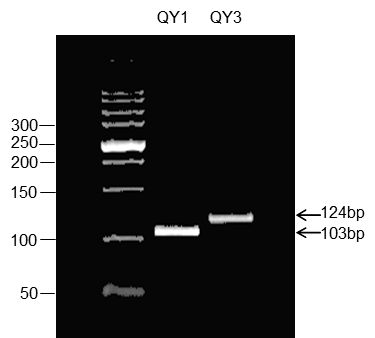


Figure S1 Development of the marker *HTpros* for amplifying the two different alleles of *HTMYB2* (QY1 and QY3). The fragments yielded by the marker were either 124bp or 103 bp, which were indicative of promoter in QY1 and QY3*,* respectively.
