# Supplementary material for: Functional MYB transcription factor gene HtMYB2 is associated with anthocyanin biosynthesis in Helianthus tuberosus L
Source: BMC Plant Biol. 2020 Jun 1;20:247. doi: 10.1186/s12870-020-02463-8 (PMC7268318; doi:10.1186/s12870-020-02463-8)
Supplement: Supplementary file 2 — Additional file 2: Table S1. The statistic of sequencing, filtering and assembling in transcriptome analysis. [file 12870_2020_2463_MOESM2_ESM.docx]

Table S1 The statistic of sequencing, filtering and assembling in transcriptome analysis

| Sample | Sequencing and Filtering | | | | Unigene Assembling | | | |
| --- | --- | --- | --- | --- | --- | --- | --- | --- |
|  | Total Raw Reads  (M) | Total Clean Reads  (M) | Total Clean Bases  (Gb) | Clean Reads Q20  (%) | Total Number | Total Length | Mean Length | N50 |
| white1 | 55.54 | 55.52 | 8.33 | 96.75 | 104278 | 94491519 | 906 | 1339 |
| white2 | 65.44 | 65.43 | 9.81 | 96.53 | 109198 | 98634405 | 903 | 1327 |
| white3 | 66.35 | 66.33 | 9.95 | 96.72 | 107669 | 98650570 | 916 | 1361 |
| purple1 | 40.04 | 40.03 | 6 | 96.52 | 85919 | 75605924 | 879 | 1318 |
| purple2 | 55.74 | 55.73 | 8.36 | 96.82 | 100405 | 87834032 | 874 | 1297 |
| purple3 | 50.61 | 50.6 | 7.59 | 96.83 | 96399 | 84890351 | 880 | 1319 |
| Total | 333.72 | 333.64 | 50.04 | 580.17 | 197769 | 2.26E+08 | 1140 | 1762 |
