# Supplementary material for: Functional MYB transcription factor gene HtMYB2 is associated with anthocyanin biosynthesis in Helianthus tuberosus L
Source: BMC Plant Biol. 2020 Jun 1;20:247. doi: 10.1186/s12870-020-02463-8 (PMC7268318; doi:10.1186/s12870-020-02463-8)
Supplement: Supplementary file 3 — Additional file 3: Table S2. The expression level of structural and regulation genes in the anthocyanin biosynthesis pathway. [file 12870_2020_2463_MOESM3_ESM.docx]

Table S2 The expression level of structural and regulation genes in the anthocyanin biosynthesis pathway

| Gene | Gene ID | Length | White-Expression | Purple-Expression | log2FoldChange | Regulation | Log2FoldChange  (Average) |
| --- | --- | --- | --- | --- | --- | --- | --- |
| MYB | Unigene44371_All | 980 | 0.534666333 | 144.0621449 | 8.073836737 | Up | 2.69 |
| bHLH | Unigene58060_All | 1677 | 15.6361371 | 20.63267112 | 0.400046466 | * | 0.04 |
|  | CL4512.Contig5_All | 2625 | 53.41380063 | 129.0148085 | 1.272252222 | Up |  |
|  | CL4512.Contig4_All | 2722 | 92.08882081 | 203.3894165 | 1.143146675 | Up |  |
|  | CL4512.Contig2_All | 2430 | 99.34126695 | 167.2732546 | 0.751741739 | * |  |
|  | CL4512.Contig3_All | 2564 | 73.00730083 | 232.3876641 | 1.67042084 | Up |  |
|  | CL4512.Contig1_All | 2305 | 53.4448338 | 96.12690126 | 0.84688973 | * |  |
|  | Unigene27918_All | 1244 | 36.11493123 | 0.65963871 | -5.774775549 | Down |  |
| CHS | Unigene33222_All | 1431 | 0.982869187 | 12829.22257 | 13.67207481 | Up | 3.98 |
|  | Unigene42101_All | 1347 | 41.31596874 | 0.786157245 | -5.715737781 | Down |  |
| CHI | CL2784.Contig1_All | 624 | 21.56856674 | 184.5594574 | 3.097083452 | Up | 0.18 |
|  | Unigene53900_All | 667 | 67.60055667 | 344.9028507 | 2.351083021 | Up |  |
|  | Unigene5139_All | 4262 | 17.98198555 | 77.76250974 | 2.112522451 | Up |  |
|  | Unigene18545_All | 2086 | 11.3755923 | 39.74977762 | 1.805005124 | Up |  |
|  | CL2784.Contig3_All | 889 | 142.9761097 | 366.3472825 | 1.357437811 | Up |  |
|  | Unigene19203_All | 4394 | 77.43588968 | 180.8543982 | 1.223754402 | Up |  |
|  | CL10506.Contig1_All | 724 | 129.50532 | 277.5834164 | 1.099910016 | Up |  |
|  | Unigene11669_All | 1910 | 108.5883954 | 239.7430379 | 1.142618986 | Up |  |
|  | Unigene10172_All | 3741 | 238.5607276 | 45.69990215 | -2.384093581 | Down |  |
|  | Unigene7050_All | 1709 | 53.41027359 | 12.01101753 | -2.152758898 | Down |  |
|  | Unigene19638_All | 1311 | 56.79672944 | 14.20450018 | -1.999459789 | Down |  |
| F3H | CL9203.Contig4_All | 2346 | 14.36471027 | 959.6381478 | 6.061889709 | Up | 5.49 |
|  | Unigene14414_All | 1490 | 165.4213703 | 4180.693352 | 4.659524699 | Up |  |
|  | CL9203.Contig3_All | 2497 | 5.170389474 | 46.94950844 | 3.182765188 | Up |  |
|  | CL802.Contig2_All | 949 | 58.85209064 | 170.1153314 | 1.531347596 | Up |  |
| F3'H | CL13771.Contig2_All | 491 | 0.507105041 | 48.54504433 | 6.5808956 | Up | 2.91 |
|  | CL13771.Contig5_All | 1344 | 53.23304648 | 1567.645866 | 4.880133745 | Up |  |
|  | CL1367.Contig2_All | 1702 | 2337.951 | 2029.205 | -0.20433 | * |  |
|  | CL6058.Contig3_All | 1730 | 33.7318 | 44.18951 | 0.389595 | * |  |
| F3'5'H | CL13771.Contig1_All | 2076 | 81.94535 | 1805.885 | 4.461901 | Up | 3.33 |
|  | Unigene58716_All | 1864 | 31.86027 | 612.3998 | 4.264643 | Up |  |
|  | Unigene32542_All | 1706 | 68.85538 | 167.2116 | 1.280034 | Up |  |
| DFR | CL9517.Contig40_All | 1087 | 0.621120949 | 1895.632606 | 11.57551753 | Up | 6.72 |
|  | CL9517.Contig12_All | 775 | 0.296032977 | 330.3733784 | 10.12412383 | Up |  |
|  | CL9517.Contig30_All | 2681 | 4.818661117 | 4877.011213 | 9.98314923 | Up |  |
|  | CL9517.Contig46_All | 353 | 0.24406582 | 62.51878598 | 8.000875684 | Up |  |
|  | CL9517.Contig37_All | 1202 | 0.243065879 | 53.47534851 | 7.781382782 | Up |  |
|  | Unigene3110_All | 906 | 0.212864488 | 8.410910131 | 5.304254731 | Up |  |
|  | CL19063.Contig6_All | 1176 | 37.72613122 | 275.4430029 | 2.868117748 | Up |  |
|  | CL19063.Contig1_All | 1230 | 13.56097842 | 48.38099845 | 1.83497927 | Up |  |
|  | Unigene67810_All | 536 | 69.97739346 | 181.0426293 | 1.371368609 | Up |  |
| ANS | CL13383.Contig1_All | 1264 | 1.201045218 | 3621.78899 | 11.55819631 | Up | 0.25 |
|  | CL19294.Contig9_All | 1147 | 1.404919207 | 506.2432141 | 8.493199687 | Up |  |
|  | CL13383.Contig2_All | 1352 | 9.58555164 | 2641.568491 | 8.106317641 | Up |  |
|  | CL8012.Contig4_All | 1291 | 24.6921607 | 0.316360274 | -6.286340826 | Down |  |
|  | Unigene97055_All | 754 | 10.92264863 | 0.283816098 | -5.266222505 | Down |  |
|  | CL7075.Contig4_All | 603 | 9.69926883 | 0.275840284 | -5.135970923 | Down |  |
|  | CL20031.Contig6_All | 1757 | 54.42114188 | 1.988164088 | -4.774658492 | Down |  |
|  | CL7075.Contig2_All | 1511 | 55.93019257 | 2.135257884 | -4.711145072 | Down |  |
