# Supplementary material for: Functional MYB transcription factor gene HtMYB2 is associated with anthocyanin biosynthesis in Helianthus tuberosus L
Source: BMC Plant Biol. 2020 Jun 1;20:247. doi: 10.1186/s12870-020-02463-8 (PMC7268318; doi:10.1186/s12870-020-02463-8)
Supplement: Supplementary file 4 — Additional file 4: Table S3. The promoter prediction of HtMYB2. [file 12870_2020_2463_MOESM4_ESM.docx]

Table S3 The promoter prediction of *HtMYB2*

| Sample | Start | End | Score | Promoter Sequence |
| --- | --- | --- | --- | --- |
| QY1 | -1336 | -1286 | 0.81 | TTTTTTTCTCTATATAAGTATAGCAATATGTTACTCATATTAAAGATAAA |
|  | -1300 | -1250 | 0.83 | ATATTAAAGATAAAAAATGCTCTTATGGTGTAATTTAAAAAAAAAAGTTA |
|  | -1239 | -1189 | 1.00 | TTATTGACGTTTAAAAAAGACGGGGGGAAGTTACATGTGCATTACCTAAT |
| QY3 | -1339 | -1289 | 0.81 | TTTTTTTCTCTATATAAGTATAGCAATATGTTACTCATATTAAAGATAAA |
|  | -1238 | -1188 | 1.00 | TATTGACGTTTAAAAAAGACGGGGGGGAAGTTACATGTGCATTACCTAAT |
