# Supplementary material for: Functional MYB transcription factor gene HtMYB2 is associated with anthocyanin biosynthesis in Helianthus tuberosus L
Source: BMC Plant Biol. 2020 Jun 1;20:247. doi: 10.1186/s12870-020-02463-8 (PMC7268318; doi:10.1186/s12870-020-02463-8)
Supplement: Supplementary file 5 — Additional file 5: Table S4. The origin of the Helianthus tuberosus and their MYB genotype examined using HtproS marker. [file 12870_2020_2463_MOESM5_ESM.docx]

Table S4. The origin of the Helianthus tuberosus and their MYB genotype examined using HtproS marker

| Item | voucher number | Source | Phenotype | Genotype | Used in Figure 5B |
| --- | --- | --- | --- | --- | --- |
| 1 | QHYYS-OO78 | China | Purple | HtMYB2-QY1 | √ |
| 2 | QHYYS-OO79 | China | Purple | HtMYB2-QY1 |  |
| 3 | QHYYS-OO80 | China | Purple | HtMYB2-QY1 | √ |
| 4 | QHYYS-OO81 | China | Purple | HtMYB2-QY1 | √ |
| 5 | QHYYS-OO82 | China | Purple | HtMYB2-QY1 | √ |
| 6 | QHYYS-OO83 | China | Purple | HtMYB2-QY1 | √ |
| 7 | QHYYS-OO84 | China | Purple | HtMYB2-QY1 |  |
| 8 | QHYYS-OO85 | China | Purple | HtMYB2-QY1 |  |
| 9 | QHYYS-OO86 | China | Purple | HtMYB2-QY1 | √ |
| 10 | QHYYS-OO87 | China | Purple | HtMYB2-QY1 |  |
| 11 | QHYYS-OO88 | China | Purple | HtMYB2-QY1 | √ |
| 12 | QHYYS-OO89 | China | Purple | HtMYB2-QY1 |  |
| 13 | QHYYS-OO90 | China | Purple | HtMYB2-QY1 |  |
| 14 | QHYYS-OO91 | China | Purple | HtMYB2-QY1 |  |
| 15 | QHYYS-OO92 | China | Purple | HtMYB2-QY1 | √ |
| 16 | QHYYS-OO93 | China | Purple | HtMYB2-QY1 |  |
| 17 | QHYYS-OO94 | China | Purple | HtMYB2-QY1 |  |
| 18 | QHYYS-OO95 | China | Purple | HtMYB2-QY1 | √ |
| 19 | QHYYS-OO96 | China | Purple | HtMYB2-QY1 | √ |
| 20 | QHYYS-OO97 | China | Purple | HtMYB2-QY1 | √ |
| 21 | QHYYS-OO98 | China | Purple | HtMYB2-QY1 |  |
| 22 | QHYYS-OO99 | China | Purple | HtMYB2-QY1 |  |
| 23 | QHYYS-OO100 | China | Purple | HtMYB2-QY1 |  |
| 24 | QHYYS-OO101 | China | Purple | HtMYB2-QY1 |  |
| 25 | QHYYS-OO102 | China | Purple | HtMYB2-QY1 |  |
| 26 | QHYYS-OO103 | China | Purple | HtMYB2-QY1 |  |
| 27 | QHYYS-OO104 | China | Purple | HtMYB2-QY1 |  |
| 28 | QHYYS-OO105 | China | Purple | HtMYB2-QY1 |  |
| 29 | QHYYS-OO106 | China | Purple | HtMYB2-QY1 |  |
| 30 | QHYYS-OO107 | China | Purple | HtMYB2-QY1 |  |
| 31 | QHYYS-OO108 | China | Purple | HtMYB2-QY1 |  |
| 32 | QHYYS-OO109 | China | Purple | HtMYB2-QY1 |  |
| 33 | QHYYS-OO110 | China | Purple | HtMYB2-QY1 |  |
| 34 | QHYYS-OO111 | China | Purple | HtMYB2-QY1 |  |
| 35 | QHYYS-OO112 | China | Purple | HtMYB2-QY1 |  |
| 36 | QHYYS-OO113 | China | Purple | HtMYB2-QY1 |  |
| 37 | QHYYS-OO114 | China | Purple | HtMYB2-QY1 |  |
| 38 | QHYYS-OO115 | China | Purple | HtMYB2-QY1 |  |
| 39 | QHYYS-OO116 | China | Purple | HtMYB2-QY1 |  |
| 40 | QHYYS-OO117 | China | Purple | HtMYB2-QY1 |  |
| 41 | QHYYS-OO118 | China | Purple | HtMYB2-QY1 |  |
| 42 | QHYYS-OO119 | China | Purple | HtMYB2-QY1 |  |
| 43 | QHYYS-OO120 | China | Purple | HtMYB2-QY1 |  |
| 44 | QHYYS-OO121 | China | Purple | HtMYB2-QY1 |  |
| 45 | QHYYS-OO122 | China | Purple | HtMYB2-QY1 |  |
| 46 | QHYYS-OO123 | China | Purple | HtMYB2-QY1 |  |
| 47 | QHYYS-OO124 | China | Purple | HtMYB2-QY1 |  |
| 48 | QHYYS-OO125 | China | Purple | HtMYB2-QY1 |  |
| 49 | QHYYS-OO126 | China | Purple | HtMYB2-QY1 |  |
| 50 | QHYYS-OO127 | China | Purple | HtMYB2-QY1 |  |
| 51 | QHYYS-OO128 | China | Purple | HtMYB2-QY1 |  |
| 52 | QHYYS-OO129 | China | Purple | HtMYB2-QY1 |  |
| 53 | QHYYS-OO130 | China | Purple | HtMYB2-QY1 |  |
| 54 | QHYYS-OO131 | China | Purple | HtMYB2-QY1 |  |
| 55 | QHYYS-OO132 | Danmark | Purple | HtMYB2-QY1 |  |
| 56 | QHYYS-OO133 | Danmark | Purple | HtMYB2-QY1 |  |
| 57 | QHYYS-OO134 | Danmark | Purple | HtMYB2-QY1 |  |
| 58 | QHYYS-OO135 | French | Purple | HtMYB2-QY1 |  |
| 59 | QHYYS-OO136 | French | Purple | HtMYB2-QY1 |  |
| 60 | QHYYS-OO137 | French | Purple | HtMYB2-QY1 |  |
| 61 | QHYYS-OO138 | French | Purple | HtMYB2-QY1 |  |
| 62 | QHYYS-OO139 | French | Purple | HtMYB2-QY1 |  |
| 63 | QHYYS-OO140 | French | Purple | HtMYB2-QY1 |  |
| 64 | QHYYS-OO141 | French | Purple | HtMYB2-QY1 |  |
| 65 | QHYYS-OO142 | Thailand | Purple | HtMYB2-QY1 |  |
| 66 | QHYYS-OO143 | Thailand | Purple | HtMYB2-QY1 |  |
| 67 | QHYYS-OO144 | Thailand | Purple | HtMYB2-QY1 |  |
| 68 | QHYYS-OO145 | Thailand | Purple | HtMYB2-QY1 |  |
| 69 | QHYYS-OO146 | Thailand | Purple | HtMYB2-QY1 |  |
| 70 | QHYYS-OO147 | Thailand | Purple | HtMYB2-QY1 |  |
| 71 | QHYYS-OO148 | Thailand | Purple | HtMYB2-QY1 |  |
| 72 | QHYYS-OO149 | Thailand | Purple | HtMYB2-QY1 |  |
| 73 | QHYYS-OO150 | Thailand | Purple | HtMYB2-QY1 |  |
| 74 | QHYYS-OO151 | Thailand | Purple | HtMYB2-QY1 |  |
| 75 | QHYYS-OO152 | Thailand | Purple | HtMYB2-QY1 |  |
| 76 | QHYYS-OO153 | Thailand | Purple | HtMYB2-QY1 |  |
| 77 | QHYYS-OO154 | Cannada | Purple | HtMYB2-QY1 |  |
| 78 | QHYYS-OO155 | China | Purple | HtMYB2-QY1 |  |
| 79 | QHYYS-OO156 | China | Purple | HtMYB2-QY1 |  |
| 80 | QHYYS-OO157 | China | Purple | HtMYB2-QY1 |  |
| 81 | QHYYS-OO158 | China | Purple | HtMYB2-QY1 |  |
| 82 | QHYYS-OO159 | China | Purple | HtMYB2-QY1 |  |
| 83 | QHYYS-OO160 | China | Purple | HtMYB2-QY1 |  |
| 84 | QHYYS-OO161 | China | Purple | HtMYB2-QY1 |  |
| 85 | QHYYS-OO162 | China | Purple | HtMYB2-QY1 |  |
| 86 | QHYYS-OO163 | China | Purple | HtMYB2-QY1 |  |
| 87 | QHYYS-OO164 | China | Purple | HtMYB2-QY1 |  |
| 88 | QHYYS-OO165 | China | Purple | HtMYB2-QY1 |  |
| 89 | QHYYS-OO166 | China | Purple | HtMYB2-QY1 |  |
| 90 | QHYYS-OO167 | China | Purple | HtMYB2-QY1 |  |
| 91 | QHYYS-OO168 | China | White | HtMYB2-QY3 | √ |
| 92 | QHYYS-OO169 | China | White | HtMYB2-QY3 | √ |
| 93 | QHYYS-OO170 | China | White | HtMYB2-QY3 | √ |
| 94 | QHYYS-OO171 | China | White | HtMYB2-QY3 | √ |
| 95 | QHYYS-OO172 | China | White | HtMYB2-QY3 | √ |
| 96 | QHYYS-OO173 | China | White | HtMYB2-QY3 | √ |
| 97 | QHYYS-OO174 | China | White | HtMYB2-QY3 | √ |
| 98 | QHYYS-OO175 | China | White | HtMYB2-QY3 | √ |
| 99 | QHYYS-OO176 | China | White | HtMYB2-QY3 | √ |
| 100 | QHYYS-OO177 | China | White | HtMYB2-QY3 | √ |
| 101 | QHYYS-OO178 | China | White | HtMYB2-QY3 | √ |
| 102 | QHYYS-OO179 | China | White | HtMYB2-QY3 |  |
| 103 | QHYYS-OO180 | China | White | HtMYB2-QY3 |  |
| 104 | QHYYS-OO181 | China | White | HtMYB2-QY3 |  |
| 105 | QHYYS-OO182 | China | White | HtMYB2-QY3 |  |
| 106 | QHYYS-OO183 | China | White | HtMYB2-QY3 |  |
| 107 | QHYYS-OO184 | China | White | HtMYB2-QY3 |  |
| 108 | QHYYS-OO185 | China | White | HtMYB2-QY3 |  |
| 109 | QHYYS-OO186 | China | White | HtMYB2-QY3 |  |
| 110 | QHYYS-OO187 | China | White | HtMYB2-QY3 |  |
| 111 | QHYYS-OO188 | China | White | HtMYB2-QY3 |  |
| 112 | QHYYS-OO189 | China | White | HtMYB2-QY3 |  |
| 113 | QHYYS-OO190 | China | White | HtMYB2-QY3 |  |
| 114 | QHYYS-OO191 | China | White | HtMYB2-QY3 |  |
| 115 | QHYYS-OO192 | China | White | HtMYB2-QY3 |  |
| 116 | QHYYS-OO193 | China | White | HtMYB2-QY3 |  |
| 117 | QHYYS-OO194 | China | White | HtMYB2-QY3 |  |
| 118 | QHYYS-OO195 | China | White | HtMYB2-QY3 |  |
| 119 | QHYYS-OO196 | China | White | HtMYB2-QY3 |  |
| 120 | QHYYS-OO197 | China | White | HtMYB2-QY3 |  |
| 121 | QHYYS-OO198 | China | White | HtMYB2-QY3 |  |
| 122 | QHYYS-OO199 | China | White | HtMYB2-QY3 |  |
| 123 | QHYYS-OO200 | China | White | HtMYB2-QY3 |  |
| 124 | QHYYS-OO201 | China | White | HtMYB2-QY3 |  |
| 125 | QHYYS-OO202 | Denmark | White | HtMYB2-QY3 |  |
| 126 | QHYYS-OO203 | Denmark | White | HtMYB2-QY3 |  |
| 127 | QHYYS-OO204 | Denmark | White | HtMYB2-QY3 |  |
| 128 | QHYYS-OO205 | Denmark | White | HtMYB2-QY3 |  |
| 129 | QHYYS-OO206 | French | White | HtMYB2-QY3 |  |
| 130 | QHYYS-OO207 | French | White | HtMYB2-QY3 |  |
| 131 | QHYYS-OO208 | French | White | HtMYB2-QY3 |  |
| 132 | QHYYS-OO209 | French | White | HtMYB2-QY3 |  |
| 133 | QHYYS-OO210 | French | White | HtMYB2-QY3 |  |
| 134 | QHYYS-OO211 | French | White | HtMYB2-QY3 |  |
| 135 | QHYYS-OO212 | French | White | HtMYB2-QY3 |  |
| 136 | QHYYS-OO213 | French | White | HtMYB2-QY3 |  |
| 137 | QHYYS-OO214 | French | White | HtMYB2-QY3 |  |
| 138 | QHYYS-OO215 | French | White | HtMYB2-QY3 |  |
| 139 | QHYYS-OO216 | Thailand | White | HtMYB2-QY3 |  |
| 140 | QHYYS-OO217 | French | White | HtMYB2-QY3 |  |
| 141 | QHYYS-OO218 | Thailand | White | HtMYB2-QY3 |  |
| 142 | QHYYS-OO219 | Thailand | White | HtMYB2-QY3 |  |
| 143 | QHYYS-OO220 | Thailand | White | HtMYB2-QY3 |  |
| 144 | QHYYS-OO221 | Russia | White | HtMYB2-QY3 |  |
| 145 | QHYYS-OO222 | Canada | White | HtMYB2-QY3 |  |
| 146 | QHYYS-OO223 | Canada | White | HtMYB2-QY3 |  |
| 147 | QHYYS-OO224 | U.S.A | White | HtMYB2-QY3 |  |
| 148 | QHYYS-OO225 | U.S.A | White | HtMYB2-QY3 |  |
| 149 | QHYYS-OO226 | U.S.A | White | HtMYB2-QY3 |  |
| 150 | QHYYS-OO227 | Hungary | White | HtMYB2-QY3 |  |
| 151 | QHYYS-OO228 | Hungary | White | HtMYB2-QY3 |  |
| 152 | QHYYS-OO229 | China | White | HtMYB2-QY3 |  |
| 153 | QHYYS-OO230 | China | White | HtMYB2-QY3 |  |
| 154 | QHYYS-OO231 | China | White | HtMYB2-QY3 |  |
| 155 | QHYYS-OO232 | China | White | HtMYB2-QY3 |  |
| 156 | QHYYS-OO233 | China | White | HtMYB2-QY3 |  |
| 157 | QHYYS-OO234 | China | White | HtMYB2-QY3 |  |
| 158 | QHYYS-OO235 | China | White | HtMYB2-QY3 |  |
| 159 | QHYYS-OO236 | China | White | HtMYB2-QY3 |  |
| 160 | QHYYS-OO237 | China | White | HtMYB2-QY3 |  |
| 161 | QHYYS-OO238 | China | White | HtMYB2-QY3 |  |
| 162 | QHYYS-OO239 | China | White | HtMYB2-QY3 |  |
| 163 | QHYYS-OO240 | China | White | HtMYB2-QY3 |  |
| 164 | QHYYS-OO241 | China | White | HtMYB2-QY3 |  |
| 165 | QHYYS-OO242 | China | White | HtMYB2-QY3 |  |
| 166 | QHYYS-OO243 | China | White | HtMYB2-QY3 |  |
| 167 | QHYYS-OO244 | China | White | HtMYB2-QY3 |  |
| 168 | QHYYS-OO245 | China | White | HtMYB2-QY3 |  |
| 169 | QHYYS-OO246 | China | White | HtMYB2-QY3 |  |
| 170 | QHYYS-OO247 | China | White | HtMYB2-QY3 |  |
| 171 | QHYYS-OO248 | China | White | HtMYB2-QY3 |  |
| 172 | QHYYS-OO249 | China | White | HtMYB2-QY3 |  |
| 173 | QHYYS-OO250 | China | White | HtMYB2-QY3 |  |
| 174 | QHYYS-OO251 | China | White | HtMYB2-QY3 |  |
| 175 | QHYYS-OO252 | China | White | HtMYB2-QY3 |  |
| 176 | QHYYS-OO253 | China | White | HtMYB2-QY3 |  |
| 177 | QHYYS-OO254 | China | White | HtMYB2-QY3 |  |
| 178 | QHYYS-OO255 | China | White | HtMYB2-QY3 |  |
| 179 | QHYYS-OO256 | China | White | HtMYB2-QY3 |  |
| 180 | QHYYS-OO257 | China | White | HtMYB2-QY3 |  |
