# Supplementary material for: Functional MYB transcription factor gene HtMYB2 is associated with anthocyanin biosynthesis in Helianthus tuberosus L
Source: BMC Plant Biol. 2020 Jun 1;20:247. doi: 10.1186/s12870-020-02463-8 (PMC7268318; doi:10.1186/s12870-020-02463-8)
Supplement: Supplementary file 6 — Additional file 6: Table S5. Oligo nucleotide primers used in this work. [file 12870_2020_2463_MOESM6_ESM.docx]

Table S5 Oligo nucleotide primers used in this work

| Primer | Sequence (5'-3') | Function |
| --- | --- | --- |
| HtMYB2cdsF | ATGAGTCAGAATTGTAATAC | Cloning HtMYB2 cDNAs |
| HtMYB2cdsR | CAGTAAAGAAGGTCCAACATGA |  |
| HtMYB2attb1 | AAAAAGCAGGCTTCATGAGTCAGAATTGTAATAC | Construction of 35S-HtMYB2 |
| HtMYB2attb2 | AGAAAGCTGGGTCTCAGTAAAGAAGGTCCAACATGA |  |
| Attb1 adapter | GGGGACAAGTTTGTACAAAAAAGCAGGCT | Universal attB adapter primers |
| Attb2 adapter | GGGGACCACTTTGTACAAGAAAGCTGGGT |  |
| Tail-PCR-R1 | TATCTAAACCTTCCGCCTCCAG | First round Tail-PCR |
| Tail-PCR-R2 | TGGGGAGATGTTGAAGGTGTTG | Second round Tail-PCR |
| Tail-PCR-R3 | TGGTAGTCTTCCCGCAATCAAT | Third round Tail-PCR。 |
| Httubulin1 | ATGTCTCCTGCATCCAAGTC | Amplifying Helianthus tuberosus tubulin gene transcripts (as internal control of qPCR) |
| Httubulin2 | GAACACCTGCGTTAGCATTG |  |
| HtMYB-RT-F | TGAGTCAGAATTGTAATACGAG |  |
| HtMYB-RT-R | ACTCTTCCTGCATCGGTTTA |  |
| HtCHS-F | ATGCTTAGCATCCAGGAGTT |  |
| HtCHS-R | TTTGGAGCACACAATTAGGC |  |
| HtCHI-F | ATGGTGATGGTGGATGATAT |  |
| HtCHI-R | GAAAATGAATCTCGATGTCG |  |
| HtF3H-F | ATGGTGATTTCAGCAAATAC |  |
| HtF3H-R | TGGTTGTGAGTTCATCTTGT |  |
| HtF3'H-F | ACCACTTTCATATACCTGAG |  |
| HtF3'H-R | CTTAGGGTTGAGAATGGTTC |  |
| HtF3'5'H-RT1 | TTGTGGATGGCGAATGAGTT |  |
| HtF3'5'H-RT2 | GGATGTCGAAATCAAAGCTT |  |
| HtDFR-F | AGGTGAAACATTTGATAGAACTACC |  |
| HtDFR-R | CCTTCAATGGTTTCATCAAA |  |
| HtANS-F | ATGGTTGAATCCCAGTCAGG |  |
| HtANS-R | ATCTTTCTGGCAGTTGGTCA |  |
| HtproS-F | CGCCACCTTAATTATAGGA | Amplifying the HTMYB2 difference in promoter |
| HtproS-R170 | TTTGTATTAAAAAAAGAGAT |  |
| NtActin-F | AATGATCGGAATGGAAGCTG | Amplifying Tobacco Actin gene transcripts (as internal control of qPCR) |
| NtActin-R | TGGTACCACCACTGAGGACA |  |
| NtCHI-F | GAAATCCTCCGATCCAGTGA |  |
| NtCHI-R | CAACGTTGACAACATCAGGC |  |
| NbCHS-F | AGAAAAGCCTTGTGGAAGCA |  |
| NbCHS-R | ACTTGGTCCAAAATTGCAGG |  |
| NtF3H-F | ACAGGGTGAAGTGGTCCAAG |  |
| NtF3H-R | CCTTGGTTAAGGCCTCCTTC |  |
| NtF3'H-F | TCCAAGAATACTGGCCCAAG |  |
| NtF3'H-R | CTCACAACTCTCGGATGCAA |  |
| NtF3'5'H-F | GCCATAGATACAAGCCTCTT |  |
| NtF3'5'H-R | ACCTAGAAGAGGAAGAGCGC |  |
| NbDFR-F | TCCCATCATGCGATCATCTA |  |
| NbDFR-R | ATGGCTTCTTTGTCACGTCC |  |
| NtANS-F | TGGCGTTGAAGCTCATACTG |  |
| NtANS-R | TTTCAAGGGTGTCCCCAATA |  |
